# Pancreatic body adenocarcinoma with neuroendocrine tumor characteristics: A case report

**DOI:** 10.3892/ol.2014.1873

**Published:** 2014-02-12

**Authors:** HIDEHIRO TAJIMA, HIROHISA KITAGAWA, MASATOSHI SHOJI, TOSHIFUMI WATANABE, SHINICHI NAKANUMA, KOICHI OKAMOTO, SEISHO SAKAI, JUN KINOSHITA, ISAMU MAKINO, HIROYUKI FURUKAWA, KEISHI NAKAMURA, HIRONORI HAYASHI, KATSUNOBU OYAMA, MASAFUMI INOKUCHI, HISATOSHI NAKAGAWARA, TOMOHARU MIYASHITA, HIROSHI ITOH, HIROYUKI TAKAMURA, ITASU NINOMIYA, SACHIO FUSHIDA, TAKASHI FUJIMURA, TETSUO OHTA, HIROHIDE SATOH, HIROKO IKEDA, KENICHI HARADA, YASUNI NAKANUMA

**Affiliations:** 1 Department of Gastroenterologic Surgery, Division of Cancer Medicine, Graduate School of Medicine Science, Kanazawa University, Kanazawa, Ishikawa 920-8641, Japan; 2 Division of Pathology, Kanazawa University Hospital, Kanazawa University, Kanazawa, Ishikawa 920-8641, Japan; 3 Department of Human Pathology, Division of Cancer Medicine, Graduate School of Medicine Science, Kanazawa University, Kanazawa, Ishikawa 920-8641, Japan

**Keywords:** pancreatic adenocarcinoma, neuroendocrine tumor, immunohistochemistry

## Abstract

A 61-year-old female with pancreatic body cancer underwent a distal pancreatectomy. The tumor was a moderately- to poorly*-*differentiated adenocarcinoma. Tumor growth filled the dilated main pancreatic duct (MPD) and infiltrated the surrounding area. Six months later, metastases to the left diaphragm and MPD of the remnant pancreatic head were detected. Chemoradiotherapy was administered, but the patient succumbed 22 months after surgery. An autopsy demonstrated that a moderately- to poorly-differentiated adenocarcinoma had arisen from the pancreatic head and infiltrated the duodenum and bile duct. Huge liver metastases and multiple peritoneal disseminations were also present. Microscopically, a portion of the tumor had a pseudo-rosette appearance in the adenocarcinoma component, while another section showed characteristics of a neuroendocrine tumor (NET) immunohistochemically. The original surgically-resected tumor also showed NET characteristics immunohistochemically. It is therefore necessary to search for NET components in pancreatic cancer with atypical growth and metastases, even when adenocarcinoma has been diagnosed histologically.

## Introduction

Pancreatic neoplasms usually display ductal, acinar or neuroendocrine differentiation. Pancreatic neuroendocrine tumors (pNETs), previously termed pancreatic endocrine neoplasms, are relatively uncommon and constitute only 5–8% of all pancreatic neoplasms. According to The World Health Organization (WHO) classification (2010), NETs are classed as NET G1 (carcinoid, mitotic count <2 per 10 high power fields (HPF) and/or ≤2% Ki67 index), NET G2 (mitotic count 2–20 per 10 HPF and/or 3–20% Ki67 index), NET G3 (neuroendocrine carcinoma, mitotic count >20 per 10 HPF and/or >20% Ki67 index), and mixed adenoneuroendocrine carcinoma (MANEC) (1,2). When neuroendocrine cells comprise >30% of the tumor, the lesion is classified as MANEC.

The current study presents an extremely rare case of pancreatic body cancer that was morphologically diagnosed as an adenocarcinoma, but with a growth and a form of recurrence that were atypical and indicated an MANEC, as NET components were detected immunohistochemically.

## Case report

**Figure 1:**
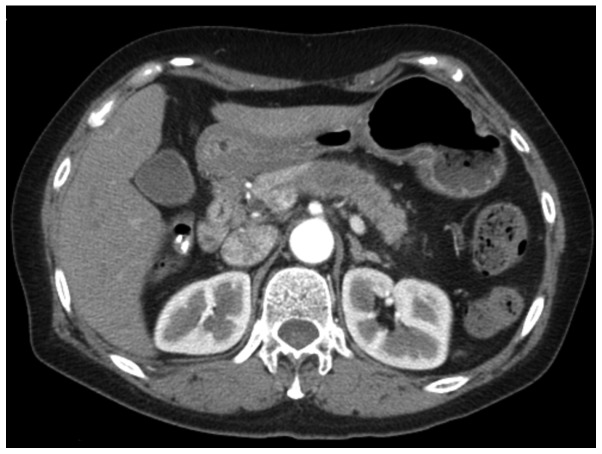
Pre-operative CT image of the pancreas. Enhanced CT showing a hypovascular tumor, 2 cm in diameter, in the pancreatic body. CT, computed tomography.

**Figure 2:**
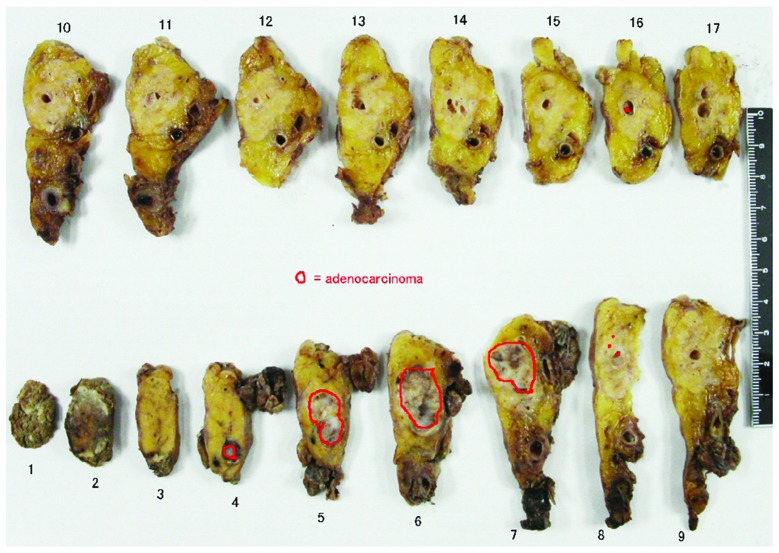
Cut-out view of the resected specimen. An adenocarcinoma is indicated by the red outline. The main tumor is observable in sections 4 to 8. A piece of tumor tissue was found in the MPD on the tail side away from the main tumor in section 16. MPD, main pancreatic duct.

**Figure 3:**
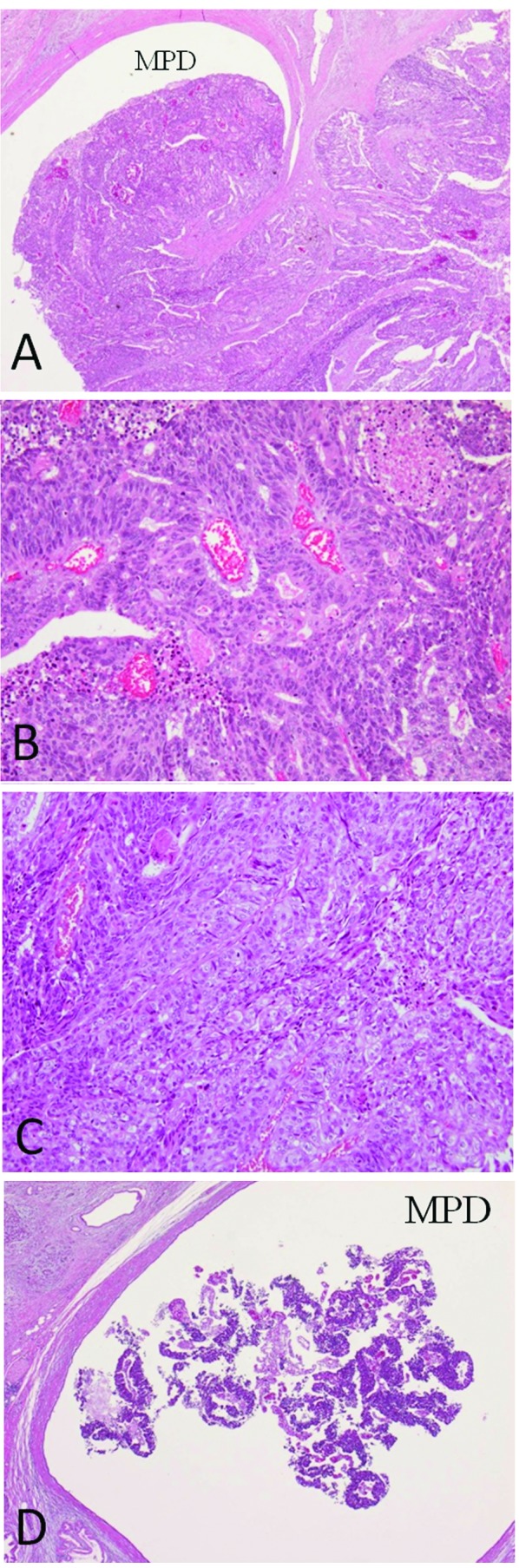
Microscopic analysis of the resected specimen by HE staining.(A). Tumor growth filling the dilated MPD and infiltrating the surrounding area. (B) Moderately- and (C) poorly-differentiated adenocarcinoma components. (D) A piece of tumor tissue was found in the MPD on the tail side away from the main tumor. HE, hematoxylin and eosin; MPD, main pancreatic duct.

The patient was a 61-year-old female found to have a tumor (2 cm in diameter) in the pancreatic body during ultrasonography, which was performed for the purpose of monitoring chronic hepatitis B. The patient was referred to Kanazawa University Hospital (Kanazawa, Japan) with a presumed diagnosis of pancreatic body cancer based on computed tomography (CT) imaging (Fig. 1). Following pre-operative chemotherapy with gemcitabine (GEM; 800 mg/m^2^) and oral S-1 (30 mg/m^2^), as previously reported (3), the patient underwent a pancreatic body and tail resection with common hepatic and celiac artery resection, also known as Appleby’s operation. Upon microscopic analysis, the tumor was diagnosed as a moderately- to poorly-differentiated adenocarcinoma. Tumor growth filled the dilated main pancreatic duct (MPD) and infiltrated the surrounding area, but neither serosal nor retroperitoneal invasion were detected, and there were no lymph node metastases (Figs. 2 and 3). Furthermore, necrosis was observed in 1/4 to 1/3 of the tumor cells and was considered to reflect the low efficacy of pre-operative chemotherapy-compatible grade IIA tumors, as judged by the Evans staging system (4). A piece of tumor tissue was found in the MPD on the tail side away from the main tumor, but it was diagnosed as an artifact at the time of surgery. The pathological diagnosis was T2, N0, M0, Stage IB, with an R0 resection, and the post-operative course was uneventful.

**Figure 4:**
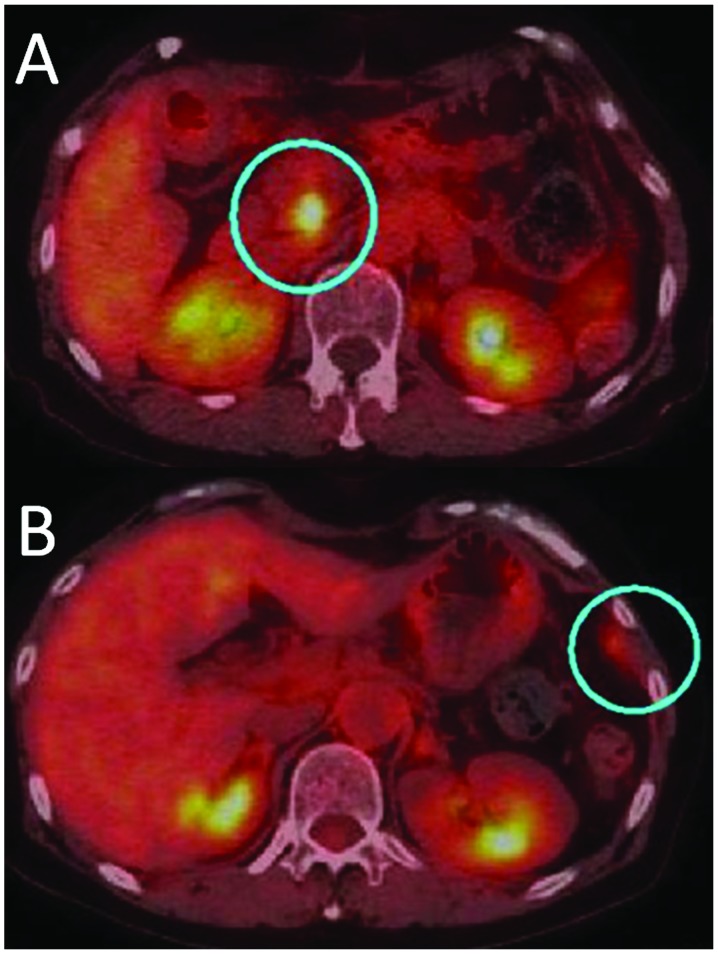
Positron emission tomography with 18-fluorodeoxyglucose (FDG-PET) findings. Blue circles reveal the abnormal uptake in the (A) pancreatic head and (B) left diaphragm.

**Figure 5:**
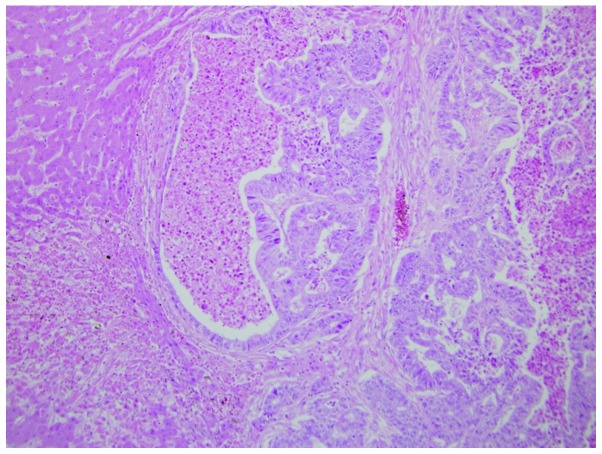
Histopathological features of liver metastasis at autopsy. Moderately- to poorly-differentiated adenocarcinoma components with necrosis were detected.

**Figure 6:**
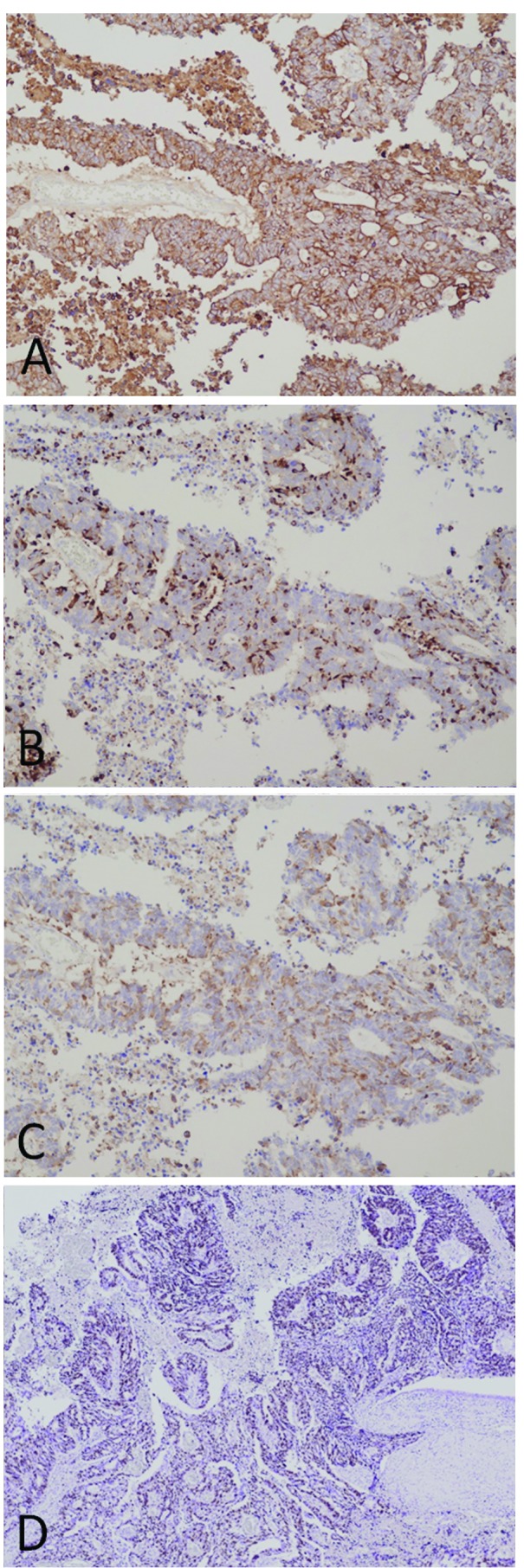
Immunohistochemical analysis of the resected pancreatic tumor. (A) Cytokeratin-7, a marker for adenocarcinomas of the pancreas, was strongly positive in nearly all the tumor cells. In the same lesion, neuroendocrine markers, (B) chromogranin A and (C) synapthophysin, were positive. (D) The Ki67 index was particularly high.

CT and positron emission tomography with 18-fluorodeoxyglucose (FDG-PET) performed 6 months later revealed metastasis to the left diaphragm and near the MPD in the remaining pancreatic head (Fig. 4). The patient underwent GEM and oral S-1 chemoradiotherapy, with the same ergimen as with the preoperative therapy with 50 Gy radiation to the pancreatic head, but liver metastases appeared 8 months later. Subsequently, the chemotherapy regimen was changed to docetaxel (30 mg/m^2^, biweekly), but the effect was minimal and the patient succumbed 22 months after the surgery. An autopsy demonstrated that a moderately- to poorly-differentiated adenocarcinoma had arisen from the pancreatic head and infiltrated the duodenum and bile duct. Furthermore, huge liver metastases formed a subphrenic abscess and there were multiple peritoneal disseminations (Fig. 5). Two distinct portions were identified microscopically; one portion had a pseudo-rosette appearance with adenocarcinoma components, while the other portion showed immunohistochemical characteristics consistent with a NET, possibly an adenoneuroendocrine carcinoma. The original surgically-resected tumor also exhibited adenocarcinoma and NET characteristics immunohistochemically. Cytokelatin-7, a marker for pancreatic adenocarcinoma, is positive in almost all tumor cells. Additionally, in the same lesion, neuroendocrine markers, chromogranin A and synapthophysin, were positive. Futhermore, the Ki67 index was particularly high (Fig. 6). There is a possibility, given the recurrence in the MPD of the remaining pancreatic head and non-contiguous main tumor growth, that this was either a caudal pancreatic duct tumor, often pathologically diagnosed for the first time, or a transitional lesion demonstrating a specific form of growth. Written informed consent was obtained from the patient’s family.

## Discussion

Neuroendocrine neoplasms, including carcinoid tumors, are commonly found in several organs, including the pancreas and gastrointestinal tract. The World Health Organization (WHO) classification (2000) (2) has been widely used to categorize NETs for all anatomical sites, and these tumors are histologically and biologically classified as well-differentiated NETs (low-grade malignancy), poorly-differentiated NETs (high-grade malignancy) and MANECs. The 2010 WHO classification strengthened the understanding of three tumor components; heterogeneity, differentiation and malignancy.

MANECs are rare and by definition are those neoplasms in which each component represents ≥30% of the lesion (1,2). In the 2010 WHO classification of tumors of the digestive tract, mixed exocrine-neuroendocrine carcinomas were defined as MANECs. They are morphologically recognizable as gland-forming epithelial and neuroendocrine neoplasms, and defined as carcinomas, since their components are histologically malignant. Notably, adenocarcinomas with scattered neuroendocrine cells, revealed by immunohistochemistry, cannot be categorized as MANECs nor neuroendocrine neoplasms with a focal non-neuroendocrine component. In such cases, almost all the tumor cells, which were recognized as adenocarcinoma morphologically, had NEC characteristics immunohistochemically.

For the treatment of unresectable pNETs, the National Comprehensive Cancer Network (NCCN) guidelines recommend everolimus, sunitinib, hepatic regional therapy, cytoreductive surgery, octreotide and cytotoxic chemotherapeutic agents, including capecitabine, dacarbazine, doxorubicin, 5-FU, streptozotocin and temozolomide. On the other hand, for poorly-differentiated NECs of the pancreas, the NCCN guidelines and North American Neuroendocrine Tumor Society consensus guidelines (6) recommend combined chemotherapy with cisplatin and etoposide, one of the standard regimens employed for the treatment of small cell lung cancer. In addition, the efficacies of GEM and oral S-1 (7), bevacizumab (8), folinic acid, fluorouracil and irinotecan (9), valproic acid (10) and other treatments have also been reported.

For the present patient, GEM plus S-1 was administered pre-operatively, and a modest histopathological effect was observed. However, this regimen and the use of docetaxel were ineffective for the recurrent lesions. Therefore, there is a possibility that the necrosis observed in the resected specimen reflected properties of the tumor rather than the effects of pre-operative chemotherapy. Moreover, a small solitary tumor of the distal MPD, diagnosed as an artifact of the resected specimen, was recognized as a metastasis in retrospect. Had the tumor been shown to contain NEC components antemortem, alternative drugs could have been administered. In conclusion, it is necessary to search for the presence of NET characteristics in cases of pancreatic adenocarcinoma with a non-specific form of growth and/or an unusual clinical course. If the tumor contains NET components, there is a possibility of using other drugs for treatment.

## References

[1] Rindi G, Klimstra DS, Arnold R, Bosman FT, Carneiro F, Hruban RH, Theise ND (2010). Nomenclature and classification of neuroendocrine neoplasms of the digestive system. WHO Classification of Tumours of the Digestive System.

[2] Komminoth P, Arnold R, Capella C, Bosman FT, Carneiro F, Hruban RH, Theise ND (2010). Neuroendocrine neoplasms of the gallbladder and extrahepatic bile duct. WHO Classification of Tumours of the Digestive System.

[3] Tajima H, Ohta T, Kitagawa H (2012). Pilot study of neoadjuvant chemotherapy with gemcitabine and oral S-1 for resectable pancreatic cancer. Exp Ther Med.

[4] Evans DB, Rich TA, Byrd DR (1992). Preoperative chemoradiation and pancreaticoduodenectomy for adenocarcinoma of the pancreas. Arch Surg.

[5] Capella C, Solcia E, Sobin LH, Arnold R, Hamilton SR, Aaltonen LA (2000). Endocrine tumours of the gallbladder and extrahepatic bile ducts. WHO Classification of Tumours of the Digestive System.

[6] Strosberg JR, Coppola D, Klimstra DS, North American Neuroendocrine Tumor Society (NANETS) (2010). The NANETS consensus guidelines for the diagnosis and management of poorly differentiated (high-grade) extrapulmonary neuroendocrine carcinomas. Pancreas.

[7] Yamamoto M, Miyagawa K, Hiura M (2012). Poorly differentiated neuroendocrine carcinoma of the pancreas responsive to combination therapy with gemcitabine and S-1. Internal Med.

[8] Kasuya K, Nagakawa Y, Suzuki M (2011). Anti-vascular endothelial growth factor antibody single therapy for pancreatic neuroendocrine carcinoma exhibits a marked tumor growth-inhibitory effect. Exp Ther Med.

[9] Brixi-Benmansour H, Jouve JL, Mitry E (2011). Phase II study of first-line FOLFIRI for progressive metastatic well-differentiated pancreatic endocrine carcinoma. Dig Liver Dis.

[10] Mohammed TA, Holen KD, Jaskula-Sztul R (2011). A phase II study of valproic acid for treatment of low-grade neuroendocrine carcinoma. Oncologist.

